# Spironolactone for the treatment of acne in women, a retrospective study of 110 patients^☆^^☆☆^^★^

**DOI:** 10.1016/j.ijwd.2016.12.002

**Published:** 2017-03-13

**Authors:** J.W. Charny, J.K. Choi, W.D. James

**Affiliations:** Department of Dermatology, University of Pennsylvania, Philadelphia, PA

**Keywords:** acne, spironolactone, comprehensive acne severity scale, CASS

## Abstract

**Background:**

There is limited evidence on the safety and efficacy of spironolactone in the treatment of women with acne. Thus, for many dermatologists spironolactone remains an alternative rather than a mainstay treatment for female patients with acne.

**Methods:**

An electronic medical records search tool was used to select data from a group of women who received spironolactone to treat acne and were evaluated with the comprehensive acne severity scale (CASS) before treatment and at all follow-up visits. Data points were collected for CASS scores at each follow-up visit, concurrent and previous treatments, and side effects. These data points were used to draw conclusions about the safety and efficacy of spironolactone in this patient population.

**Results:**

There were 110 patients that met all eligibility requirements. Of these, 94 patients saw an improvement in their CASS score and 61 patients completely cleared their score to 0. There were 16 patients who did not improve and six who relapsed after initial improvement. The women saw an average improvement in their acne by 73.1% for the face, 75.9% for the chest, and 77.6% for the back. Fifty-one women experienced side effects, but only six found them bothersome enough to stop taking spironolactone.

**Conclusion:**

A majority of women in this study saw a dramatic improvement in their acne while treated with spironolactone. There were low rates of relapse or discontinuation of the medication. To further promote the use of spironolactone as a first-line systemic treatment for women with acne, there must be more prospective controlled trials.

## Introduction

Acne vulgaris is a common, treatable dermatologic disease that can cause scarring, disfigurement, and socio-psychological distress. Current treatment guidelines generally recommend topical treatments for mild acne or a combination of topical and systemic treatments for moderate-to-severe acne (Zaenglein et al., 2016). Systemic treatments fall into three groups and each targets a mechanism of acne pathophysiology: antibiotic medications, hormonal therapy, and isotretinoin. For women whose acne failed to improve with topical treatments, hormonal therapies in the form of spironolactone and/or combined oral contraceptive pills have become an important treatment strategy.

Spironolactone is a mineralocorticoid receptor antagonist that is currently indicated for the treatment of primary hyperaldosteronism, congestive heart failure, cirrhosis, nephrotic syndrome, essential hypertension, hypokalemia, and edema of pregnancy (FDA, 2014). The safety of long-term spironolactone use is well established given that it has been approved by the U.S. Food and Drug Administration (FDA) since 1960. Furthermore, the groundbreaking Randomized Aldactone Evaluation Study has shown that spironolactone definitively reduces morbidity and mortality in patients with heart failure (Pitt et al., 1999). It is also an androgen receptor antagonist and has been shown to reduce sebum production in vivo (Goodfellow et al., 1984), leading to an increase in off-label usage for the treatment of hyperandrogenism. Because androgens mediate increased sebum production, they have been implicated in the pathophysiology of acne (Zouboulis et al., 1994), which led to the current acceptance of spironolactone as a non-antibiotic alternative to traditional systemic treatments for women with acne.

Spironolactone is generally well tolerated in women and its potentially most worrisome side effects, hyperkalemia and increased risk of cancer, are not sufficiently supported by evidence. Common side effects of long-term use of spironolactone during treatment for acne include irregular menstruation, urinary frequency, dizziness, headaches, nausea, vomiting, breast tenderness, and breast enlargement (Shaw and White, 2002). Male patients who take spironolactone often experience gynecomastia, loss of libido, and general feminization that results in the termination of treatment (Hughes and Cunliffe, 1988). Therefore, men are generally not prescribed spironolactone for the treatment of acne.

Because of its anti-androgenic effects, spironolactone has been hypothesized to be associated with an increased risk of estrogen-sensitive cancers but there is currently no evidence to support this in human subjects (Biggar et al., 2013, Mackenzie et al., 2012). Additionally, a recent study investigated hyperkalemia as a potential complication of this potassium-sparing diuretic in healthy young women with acne who are the typical demographic for off-label spironolactone use (Plovanich et al., 2015). With the exclusion of women with heart failure or kidney disease, this study showed that serum potassium levels were not found to be elevated above those of the controls and it was concluded that routine potassium monitoring is unnecessary for this relatively young and healthy cohort. Spironolactone is classified as a Pregnancy Category C drug due to its association with feminization of male fetuses in animal studies (FDA, 2014). Without the need for regular blood testing or the risk of severe teratogenicity, spironolactone is an attractive alternative to treatment with isotretinoin.

The safety profile of spironolactone is better established than its efficacy in the treatment of acne. Since the characterization of its anti-androgenic effects, there have been two important randomized placebo-controlled, double blind studies of spironolactone for the treatment of patients with acne. The studies had only 36 and 21 subjects, respectively, but showed a statistically significant improvement in acne (Goodfellow et al., 1984, Muhlemann et al., 1986). A larger retrospective study showed that 93.4% of 85 patients had some amount of improvement in their acne when treated with spironolactone (Shaw, 2000). In an effort to reduce systemic effects, physicians have also begun testing the efficacy of topical spironolactone gels. One early study showed that there was no reduction in sebum excretion (Walton et al., 1986). Recent randomized controlled trials have shown mixed results in the improvement of acne, which indicates that topical spironolactone gel is not an effective alternative for systemic spironolactone (Afzali et al., 2012, Kelidari et al., 2016).

Although current evidence demonstrates the effectiveness of oral spironolactone for the treatment of acne, the studies are too few and small to prompt FDA approval or a recommendation for use by the Cochrane Database of Systemic Reviews (Brown et al., 2009). Thus, for many dermatologists spironolactone remains an alternative rather than a mainstay treatment for female patients with acne. There is a need for further research for spironolactone to gain legitimacy as a systemic acne medication. The addition of this larger retrospective study to the literature will contribute further to the ever-clearer picture of spironolactone as a safe and effective treatment for patients with acne.

## Methods

In recent years, providers at the University of Pennsylvania have evaluated patients with acne using the comprehensive acne severity scale (CASS) as an acne grading system. A CASS score of 0 (no or barely visible acne lesions) to 5 (highly inflammatory lesions with nodules and cysts) is assigned separately for the face, chest, and back on the basis of the severity of the acne in that area. All patient records at the University of Pennsylvania Health System were accessed with PennSeek, an electronic medical record search tool, to select data from those patients with the terms acne, CASS, and spironolactone in their medical records. From this group, patients who were over the age of 12 years, treated with spironolactone for acne after January 1, 2007, and evaluated with CASS scores at both the initial and follow-up appointments by one of two specific dermatology providers were selected as eligible for the study.

These patients’ medical records were thoroughly reviewed with data points inputted into a data collection sheet. The data points included age, race, height, weight, body mass index (BMI), previous topical and systemic treatments, CASS scores at the initial and follow-up visits for the face, chest, and back, spironolactone dose, side effects, and discontinuation and relapse reasons. Data tables were constructed to show the percentage of women who discontinued or relapsed and their reasons, the prevalence of side effects, the frequencies of all prior and concurrent treatments, and the percentage of women with improvement in both total and body-site specific CASS. No advanced statistical analysis was performed on the data; therefore, the collection sheet and tables served as the primary sources to draw conclusions.

## Results

There were 4,621,497 patients in the PennSeek database of which 464 patients had the terms acne, CASS, and spironolactone in their medical records. Of these patients, 127 were over the age of 12 years, treated with spironolactone for acne after January 1, 2007, and evaluated with CASS scores at both the initial and follow-up appointments by one of two specific dermatology providers. An additional 17 patients were excluded from the study due to medication non-compliance (12 patients), running out of medication (4 patients), or lack of timely follow-up (1 patient). Of the 110 remaining patients, there were 70 patients (63.6%) who were Caucasian, 7 patients (6.4%) were African American, 13 patients (11.8%) were Asian, and 10 patients (9.1%) were of other and 10 (9.1%) of unknown race. The median age was 27 years and the average BMI was 23.03.

Previous treatments, which were tried both separately and in combination, included benzoyl peroxide, topical clindamycin, topical retinoids, intralesional kenalog, systemic antibiotic medications, oral contraceptive pills, and isotretinoin. Many patients who were already using topical acne medications or oral contraceptive pills continued their use while treated with spironolactone. There were 104 patients who used some form of concurrent therapy while taking spironolactone, 92 of which were topical medications that were already used prior to the initiation of spironolactone therapy. Of these 104 patients, 15 patients were prescribed systemic antibiotic medications concurrently either as a tapering of existent antibiotic use or as an adjunct therapy due to a lack of response to treatment with spironolactone. Thirty-five patients were taking oral contraceptive pills concurrently with spironolactone therapy, 19 of whom were already taking these although not specifically for the treatment of acne and 16 of whom began taking these as a form of birth control while treated with spironolactone. The type of oral contraceptive pill was unknown in 15 patients. Of the remaining 20 patients, 19 patients took combination estrogen/progesterone pills and 1 patient took progesterone-only pills.

Of the 110 patients, 94 patients experienced some reduction in CASS score while treated with spironolactone and 61 patients became completely clear (i.e., CASS score of 0 on the face, chest, and back). The time from initial visit to each follow-up visit varied but was on average 4 months to the first follow-up, 7 months to the second follow-up, 13 months to the third follow-up, and 17 months to the fourth follow-up visit.

Before treatment, 108 patients had CASS scores that were greater than 0 on the face. Of these, 86 patients saw a reduction in their CASS score by the time of their first follow-up visit, an additional 7 patients by the second follow-up visit, and an additional 1 patient by the fourth follow-up visit (Table 1). Before treatment, 42 patients had a CASS score that was greater than 0 on the chest. Of these, 31 patients saw a reduction in their CASS score by the time of their first follow-up visit, an additional 2 patients by the second follow-up visit, and an additional 1 patient by the fourth follow-up visit (Table 2). Before treatment, 45 patients had a CASS score that was greater than 0 on the back. Of these, 34 patients saw a reduction in their CASS score by the time of their first follow-up visit, an additional 5 patients saw a reduction by the second follow-up visit, and an additional 1 patient by the fourth follow-up visit (Table 3). There was a 73.1%, 75.9%, and 77.6% reduction in CASS scores of the face, chest, and back, respectively (Table 4).

**Table 1:** Face CASS score changes at each follow-up visit

Face CASS Score		First Follow-up Visit	Second Follow-up Visit	Third Follow-up Visit	Fourth Follow-up Visit	Total
Improvement	No.	86	7	0	1	94
	%	79.6	6.5	0.0	0.9	87.0
Cleared	No.	37	20	4	4	65
	%	34.3	18.5	3.7	3.7	60.2
No Improvement	No.	22	15	15	14	14
	%	20.4	13.9	13.9	13.0	13.0

CASS, comprehensive acne severity scale.

Note: Percentages are calculated from a total of 108 patients with initial face CASS scores greater than 0. Of the 108 patients, 94 patients’ acne improved (65 of which were completely clear) and 14 patients’ did not.

**Table 2:** Chest CASS score changes at each follow-up visit

CASS Chest		First Follow-up Visit	Second Follow-up Visit	Third Follow-up Visit	Fourth Follow-up Visit	Total
Improvement	No.	31	2	0	1	34
	%	73.8	4.8	0.0	2.4	81.0
Cleared	No.	27	4	0	2	33
	%	64.3	9.5	0.0	4.8	78.6
No Improvement	No.	11	9	9	8	8
	%	26.2	21.4	21.4	19.0	19.0

CASS, comprehensive acne severity scale.

Note: Percentages are calculated from a total of 42 patients with initial chest CASS scores greater than 0. Of the 42 patients, 34 patients’ acne improved (33 of which were completely clear) and 8 patients’ did not.

**Table 3:** Back CASS score changes at each follow-up visit.

CASS Back		First Follow-up Visit	Second Follow-up Visit	Third Follow-up Visit	Fourth Follow-up Visit	Total
Improvement	No.	34	5	0	1	40
	%	75.6	11.1	0.0	2.2	88.9
Cleared	No.	28	7	0	2	37
	%	62.2	15.6	0.0	4.4	82.2
No improvement	No.	11	6	6	5	5
	%	24.4	13.3	13.3	11.1	11.1

CASS, comprehensive acne severity scale.

Note: Percentages are calculated from a total of 45 patients with initial back CASS scores greater than 0. Of the 45 patients, 40 patients’ acne improved (37 of which were completely clear) and 5 patients’ did not.

**Table 4:** Average before and after CASS scores and percentage of improvement for women who initiated treatment with CASS score greater than 0 for the face, chest, and back

Average CASS Scores (At Treatment Initiation > 0)	Before	After	Improvement (%)
Face	2.19	0.59	73.06
Chest	1.41	0.34	75.89
Back	1.65	0.37	77.58

CASS, comprehensive acne severity scale.

Of the 101 patients (92%) who initiated a 100-mg/day dose of spironolactone, 85 patients showed an initial improvement in their acne and 40 patients became completely clear when treated with this dose. Of the remaining patients, an additional 20 patients improved and 12 patients cleared their acne when treated with 150-mg/day doses, and 10 patients improved and 3 patients cleared with 200-mg/day doses. Sixteen patients did not experience improvement when treated with spironolactone and four of these patients discontinued the medication for this reason. Six patients improved but later relapsed while treated with spironolactone. Three patients relapsed while tapering their dose of spironolactone due to good initial improvement, two patients relapsed after completing a treatment course with good results, and one patient relapsed after receiving a medroxyprogesterone acetate (Depo-Provera) injection. Concurrent treatments were similarly represented in both those patients who experienced improvement and those who did not.

There were 14 patients (12.7%) who discontinued treatment with spironolactone after a consultation with their provider: six patients due to the side effects, four patients due to lack of effectiveness, three patients due to completed course of treatment, and one patients due to pregnancy. The reported side effects that led to the treatment discontinuation were excessive sleepiness, breakthrough vaginal bleeding, urinary frequency and tachycardia, irregular periods, diffuse pruritus, and anxiety. These six women represent 11.7% of the 51 women who experienced side effects during treatment with spironolactone. In total, 34 patients experienced menstrual and 26 patients experienced non-menstrual side effects (Table 5).

**Table 5:** Side effects reported among women who were treated with spironolactone

	No. of Patients (N = 110)
**Menstrual Side Effects**	
Breakthrough bleeding/spotting	25
Amenorrhea (cessation of menses)	7
Irregular menses (varied duration between periods)	4
Oligomenorrhea (periods greater than 35 days apart)	2
**Non-menstrual Side Effects**	
Lightheadedness/dizziness	7
Breast tenderness	4
Increased Urinary Frequency	4
Weight gain	2
Worsening of restless legs	2
Sleepiness/drowsiness	2
Breast enlargement	2
Dehydration	1
Weight loss	1
Hair loss	1
Generalized pruritus	1
Fatigue	1
Decreased libido	1
Photosensitivity	1
Anxiety	1
Tachycardia	1
Metallic taste	1
Abdominal pain	1

## Discussion

The results of this study showed that acne improved in the vast majority of the women and completely cleared in a large percentage during treatment with spironolactone, which further strengthens the evidence that spironolactone is an effective treatment for women with acne. Patients showed 73.1%, 75.9%, and 77.6% improvements on the face, chest, and back, respectively, which supports that spironolactone is equally effective in treating acne in multiple areas of the body. Improvements in acne regardless of the body site occurred in 85% of patients with 55% of patients who were completely clear (CASS score of 0 in all body sites) and 26% of patients who were almost clear (maximum CASS score of 1 in one or more body sites) during treatment.

Current guidelines by the American Academy of Dermatology recommend a 3-month course of oral antibiotic medications and specifically of the tetracycline class as the initial systemic treatment for moderate-to-severe acne (Zaenglein et al., 2016). No particular tetracycline has been found to be more or less effective than any other (Garner et al., 2012). Minocycline, for example, has been studied with various methods of outcome measurement and has been found to show improvement in 51% of cases (Ozolins et al., 2004) and completely or mostly clear acne in 23.6% of cases (Stewart et al., 2006). These rates suggest that a treatment regimen of 3 months with oral antibiotic medication is often not sufficient to result in marked improvement or clearance of acne. In fact, one study found that 29% of 79,565 patients were treated with oral tetracycline-class antibiotic medications for longer than 6 months (Barbieri et al., 2016).

Comparatively, a study of patients who took oral contraceptive pills to treat acne demonstrated an improvement in 81.7% of patients with clearance or almost clearance in 48.4% of patients (Leyden et al., 2002). Other similar studies used total lesion count rather than an established severity index such as CASS scores as the primary outcome with an investigator’s global assessment, which is a subjective measure, as a secondary outcome (Lucky et al., 1997, Rosen et al., 2003, van Vloten et al., 2002). On the other hand, isotretinoin caused a complete clearance of acne in nearly all patients with one or more treatment course. Approximately 40% of patients remained clear of acne, with 40% of patients presenting with a mild relapse and 20% with a relapse that required an additional course of therapy (James, 2005). Treatment with isotretinoin, however, is not as safe as spironolactone because it has multiple, potentially serious side effects such as teratogenicity and depression. Spironolactone, which has higher percentage improvement rates compared with minocycline, percentage improvement rates that are similar to oral contraceptive pills, and a better safety profile than isotretinoin, gives women an excellent opportunity to achieve and maintain acne clearance with daily use.

In contrast with the two major clinical trials of spironolactone for the treatment of acne, this study explores the maintenance of the observed efficacy (Goodfellow et al., 1984, Muhlemann et al., 1986). Most significantly, there were no women in this study who relapsed when treated with spironolactone unless they were weaned to lower doses (five patients) or their concurrent treatments were altered (one patient relapsed after receiving a medroxyprogesterone acetate injection). On the basis of this finding, a patient should maintain a dose that is effective for at least 2 months. Once this period has passed without active disease, the risk of relapse should be addressed in patients who plan to wean or discontinue treatment.

Side effects were frequent and experienced by 51 patients (46%) but only 6 patients (5%) discontinued the medication secondary to these side effects. This indicates that those women who continued the medication despite experiencing side effects either found the side effects to be generally mild or they were outweighed by the benefits of the acne improvement. The most common side effects were breakthrough bleeding or spotting, followed by amenorrhea and lightheadedness. In clinical trials of doxycycline, esophageal erosion and photosensitivity were the most common side effects. Minocycline, on the other hand, has been associated most-commonly with side effects of lightheadedness and non-specific gastrointestinal disturbances; however, reports of lupus-like syndromes and hepatitis have also been associated with treatment (Smith and Leyden, 2005).

The chronic use of oral tetracycline antibiotic medications, which is common in the treatment of acne, has been associated with an increased risk of antibiotic resistance (Levy et al., 2003), upper respiratory tract infections (Margolis et al., 2005), and inflammatory bowel disease (Margolis et al., 2010). Spironolactone, with a similar side effect profile regardless of the duration of treatment, may be a safer treatment option than oral antibiotic medications for chronic acne treatment. Isotretinoin has more significant side effects including teratogenicity, depression, xerosis, arthralgia, hypertriglyceridemia, and elevated liver enzymes (Peck et al., 1982, Strauss et al., 1984). Without the need for registration in a mandatory national distribution program (iPledge; FDA, 2012), laboratory monitoring, and pregnancy testing, spironolactone is a safer and more convenient treatment choice than isotretinoin.

## Limitations

The study was limited in its retrospective nature and lack of a control group. Factors such as concurrent treatment, time to follow-up, dose, and duration of treatment were observed and recorded but not controlled for. Concurrent treatments may confound the observed efficacy of spironolactone. Of those patients who took oral contraceptive pills while treated with spironolactone, 52% had already been taking them and 44% initiated them to prevent pregnancy, help regulate irregular menses due to spironolactone, or as an adjunct treatment to spironolactone. Of the 15 patients who were prescribed systemic antibiotic medications while treated with spironolactone, 4 patients were tapered off of the preexisting antibiotic regimens, 10 patients initiated antibiotic medications after a trial of spironolactone alone was not effective, and 1 patients was both tapered off of a preexisting antibiotic regimen and initiated a new regimen. These patients did not take trimethoprim-sulfamethoxazole as an adjunctive treatment to spironolactone because this antibiotic medication can potentially cause hyperkalemia. No uniform blood pressure or potassium testing was conducted because this generally young, healthy patient population was screened by history to have no hypertension, cardiac, or renal disease.

A major strength of this study is its relatively large sample size of 110 patients. This sample size is comparable to that of the retrospective study conducted by Shaw (2000) that included 85 patients. By using CASS scores and only patients who were treated by two dermatologists who are trained in the use of CASS, objective measures of acne severity could be obtained and compared across time with minimal variation in the assessment between patients. The large sample size and encouraging results in terms of efficacy, relapse rate, and safety make this an important study to promote more widespread use of spironolactone in the treatment of women with acne.

## Conclusions

Spironolactone is effective to treat women with acne on the face, chest, and back and it retains its utility over the course of long-term therapy. This study reaffirms the results of previous studies in terms of effectiveness and safety but adds to the literature by showing the low rate of relapse. Future spironolactone studies should focus on evaluating both the duration and dosage of treatment because these factors are vital to the development of reliable prescribing guidelines. To further promote the use of spironolactone as a first-line systemic treatment for women with acne, more prospective controlled trials must be conducted. Studies such as these will increase our knowledge base, help determine exactly how effective spironolactone is as monotherapy, and pave the way for more widespread usage.
